# What methods are used to study the association between medication adherence trajectories, estimated with the group-based trajectory modeling (GBTM) method, and health-related outcomes?—a protocol for a systematic review

**DOI:** 10.1186/s13643-022-01971-y

**Published:** 2022-05-23

**Authors:** Victoria Memoli, Giraud Ekanmian, Carlotta Lunghi, Anne-Déborah Bouhnik, Sophie Lauzier, Line Guénette

**Affiliations:** 1 Aix Marseille Univ, INSERM, IRD, SESSTIM, ISSPAM, Cancer, Biomedicine & Society Group, Equipe Labellisée Ligue Contre le Cancer, Marseille, France; 2 Population Health and Optimal Health Practices Research Axis, CHU de Québec-Université Laval Research Center, Quebec City, Canada; 3 CISSS de Chaudière-Appalaches Research Center, Lévis, Canada; 4 Department of Health Sciences, Université du Québec à Rimouski, Lévis, Canada; 5 Faculty of Pharmacy, Université Laval, Quebec City, Canada; 6 Cancer Research Center, Université Laval, Quebec City, Canada

**Keywords:** GBTM, Group-based trajectory modeling, Medication adherence, Latent class analysis, LCA, Latent class growth analysis, LCGA, Health-related outcomes

## Abstract

**Background:**

The group-based trajectory modeling (GBTM) method is increasingly used in pharmacoepidemiologic studies to describe medication adherence trajectories over time. However, assessing the associations between these medication adherence trajectories and health-related outcomes remains challenging. The purpose of this review is to identify and systematically review the methods used to assess the association between medication adherence trajectories, estimated from the GBTM method, and health-related outcomes.

**Methods:**

We will conduct a systematic review according to the recommendations of the Cochrane handbook for systematic reviews of interventions 6.2. Results will be reported following PRISMA 2020 (Preferred Reporting Items for Systematic Reviews and Meta-analyses) recommendations. We will search in the following databases: PubMed, Embase, PsycINFO, Web of Science, CINAHL, and Cochrane Library. Two reviewers will independently select articles and extract data. Discrepancies at every step will be resolved through discussion, and consensus will be reached for all disagreed articles. A third reviewer will act as a referee if needed. We will produce tables to synthesize the modalities used to estimate medication adherence trajectories with GBTM. We will also synthesize the modalities used to assess the association between these medication adherence trajectories and health-related outcomes by identifying the types of health-related outcomes studied and how they are defined, the statistical models used, and how the medication adherence trajectories were used in these models, and the effect measure yield. We will also review the limitations and biases reported by the authors and their attempts to mitigate them. We will provide a narrative synthesis.

**Discussion:**

This review will provide a thorough exploration of the strategies and methods used in medication adherence research to estimate the associations between medication adherence trajectories, estimated with GBTM, and the different health-related outcomes. It will represent the first crucial steps toward optimizing these methods in adherence studies.

**Systematic review registration:**

Prospero CRD42021213503.

**Supplementary Information:**

The online version contains supplementary material available at 10.1186/s13643-022-01971-y.

## Background

Medication adherence, which is generally defined as the extent to which patients take their medications as prescribed, is a real challenge for healthcare professionals and patients [1]. Sub-optimal adherence may be associated with poorer health-related outcomes and higher healthcare costs, depending on the disease [2–4]. Numerous studies have been conducted to assess the prevalence of medication nonadherence, identify its determinants, and determine the impact on health-related outcomes [5]. Several measures of adherence exist (e.g., the proportion of days covered (PDC), the medication possession ratio (MPR), pill count methods, or questionnaires such as the medication adherence report scale (MARS)), [5–11] but most of these measures only summarize adherence over a definite period with a number or a percentage, ranging from a few weeks to years. However, an individual’s medication adherence may vary considerably over time, and a given summarized measure may recover situations drastically different from each other.

Studying adherence dynamics over time in a population may reflect patients’ adherence behaviors more accurately than summarizing adherence as a single average measure over time. The group-based trajectory modeling (GBTM), initially developed by Nagin et al. [12] to characterize developmental trajectories of criminal activities, is increasingly used to describe the dynamic and mutable nature of medication adherence behaviors. The GBTM method makes it possible to identify groups within a population that share similar medication adherence trajectories and behaviors over time [13–15].

The identification of individuals with similar medication adherence trajectories estimated from GBTM has been largely used to describe medication adherence and to identify determinants of medication nonadherence. In this last case, medication adherence trajectories estimated through GBTM are modeled as the dependent variable (outcome) with their potential determinants as the independent variables. However, it is also possible to use medication adherence trajectories estimated from GBTM to study their associations with health-related outcomes, such as hospitalizations, death, or any critical clinical event. For this purpose, the GBTM literature does not provide specific instructions for modeling these associations. To our knowledge, there is no software procedure, macro, or instructions developed to perform analyses combining medication adherence trajectories estimated with GBTM and the outcome in a joint model. Researchers generally proceed in two steps: (1) identifying the groups with similar medication adherence trajectories with GBTM and (2) using these medication adherence trajectories as an independent variable in any suitable model with the health-related outcomes as the dependent variable.

Estimating the association between medication adherence trajectories issued from GBTM and health-related outcomes is thus challenging. Thus, it is critical to investigate the different approaches existing in the literature, the biases, and the difficulties encountered. To our knowledge, there is no systematic review of studies that have evaluated the association between medication adherence trajectories, estimated from the GBTM method, and health-related outcomes.

This review’s aim is to identify and systematically review the methodologies used to assess the association between medication adherence trajectories, estimated with the GBTM method, and health-related outcomes. We will document the different types of study designs used, medication adherence metrics used, health-related outcomes studied, statistical models and parameters used, and the limitations acknowledged by the studies’ authors and how they were addressed.

## Methods/design

This protocol is written according to the PRISMA-P (Preferred Reporting Items for Systematic Review and Meta-analysis - for protocol) checklist [16]. The filled checklist is available in Additional file 1. The review will be conducted according to the recommendations of the Cochrane handbook for a systematic review of intervention 6.2 and will be reported following PRISMA 2020 (Preferred Reporting Items for Systematic Review and Meta-analysis) recommendations.

### Eligibility criteria

We will include all studies that estimate medication adherence trajectories with the GBTM method [17] and evaluate the associations of these trajectories with specified health-related outcomes. We will not include studies on adherence to recommendations other than drug therapy (e.g., diet or exercise). Inclusion criteria are summarized in Table 1, available in Additional file 2.

#### Population

All studies measuring medication adherence trajectories in any human population will be considered. We will not apply restrictions based on population, age, race, sex, or gender.

#### Intervention, exposure

We will consider any study in which medication adherence trajectories, estimated with the GBTM method, [17] are used as an exposure variable to analyze or estimate the association between adherence and any specified health-related outcome. Medication adherence is defined as “the extent to which patients follow the instructions given to them for prescribed treatments” [18]. It is composed of three main constructs: (1) initiation (representing the extent to which a newly prescribed treatment is undertaken), (2) persistence (representing to what extent the treatment is taken for the recommended duration), and (3) implementation (representing to what extent the treatment is taken at the recommended doses and according to the recommended schedule) [19]. We will consider all studies on medication adherence, whatever the adherence concept(s) measured.

Medication adherence trajectories define descriptive longitudinal patterns of adherence over a defined time set. They help to distinguish differences in patterns of adherence for individuals or groups of individuals over time [13]. These trajectories model the evolution of adherence measures (for example, monthly PDC) over time and allow identifying people with similar adherence behaviors [13].

#### Comparators

Depending on the study design, no comparator may be required. We will not exclude any of the studies based on the comparator.

#### Outcomes

We will allow for any health-related measure described in the study as an endpoint outcome (dependent variable) in relation to medication adherence trajectories. Health-related outcomes are defined as a result measured following an intervention (e.g., surgery, treatment) or behavior and describe a consequence of disease, treatment, or event for an individual. These health-related outcomes can be symptoms, hospitalizations, death, patient’s quality of life, participation in activities, and social roles. These outcomes allow us to measure the impact of different medication adherence trajectories on individuals [20].

#### Study types

All original studies with the following designs will be included: observational studies, randomized trials, quasi-experimental studies, and cohort or case-control studies. Conference abstracts, commentaries, letters to editors, and reviews will be excluded but retrieved to identify potentially eligible references.

#### Setting and time frame

No limit will be set for the study setting or time frame. We will retain all the original studies, including those conducted in clinical settings or those part of an intervention or a trial. Selected articles will enter the initial screening stage without a time limit for execution or publication.

### Information sources

We will search for relevant article references in the following databases: PubMed, Embase, PsycINFO, Web of Science, CINAHL, and Cochrane Library. The search strategy has been developed and adapted for each database using the most sensible approach validated by a specialized librarian at Laval University. A complete description of the applied search strategies is described in Table 2 in Additional file 2. Duplicate citations will be removed using EndNote and Covidence Solution software [21]. A final manual revision of the database will be conducted to check for remaining duplicates.

We will consider and include any additional articles not identified by our search strategy and brought to our attention by screening references of selected articles or relevant systematic reviews if they meet the inclusion criteria.

All identified studies will be compiled and kept with the full text (when needed) in a shared reference management software (i.e., EndNote) [22] and with Covidence, a web-based solution for systematic reviews [21].

#### Study selection

Article selection will be performed using the Covidence solution [21]. First, two reviewers will screen titles and abstracts independently, and articles will either be included, excluded, or categorized as unsure. Articles excluded by both reviewers will not be selected. Second, reviewers will discuss discrepancies to reach a consensus for every disagreed article. A pilot screening test will be conducted on a sample of a randomly selected 10% of the articles.

Likewise, the full text of selected articles will be reviewed independently by two reviewers, and articles will be included or excluded. The reason for exclusion will be documented. Discrepancies between the two reviewers will be resolved by discussion until an agreement is reached. A third reviewer will act as a referee if needed.

### Data extraction

We will develop an extraction grid using the Cochrane checklist of items to consider in data extraction [23]. The form will include the following elements:

#### Study and population

- Study identification, including title, corresponding author’s name and contact details, country, language, and publication date
- Study design and objectives
- Health domain, including the type of population (sex, age), diseases, and medications of interest
- Sample size

Intervention (group-based trajectory modeling)

- Medication adherence measure used, including a description of data source (e.g., adherence questionnaire, adherence measured from health database, medication electronic monitoring system), and a full definition of the variable and its operationalization (e.g., continuous or dichotomized, threshold, scale, time frame)
- Software used to model the medication adherence trajectories, parameters, selection, and adequacy of the models (e.g., link function, order, number of trajectories, statistics, and clinical criteria considered for the model selection)
- Use of the medication adherence trajectories, estimated with GBTM, as the exposure variable (e.g., group membership, inverse probability weighting).

Outcomes

- Health-related outcome definition: data source, variable definitions, and their operationalization (e.g., continuous, dichotomized, time frame)
- Approach used in modeling the health-related outcomes and the rationale
- Model used to assess the association between medication adherence trajectories and health-related outcomes and its description (e.g., linear regression, logistic regression, Cox modeling) and methods to consider missing data, lost to follow-up, and censoring

#### Limitations and bias

- Limitations and biases identified by the authors
- The way in which authors tried to mitigate identified biases

The form will be tested on a random sample of 10% of the included studies. We will contact the study authors (three attempts, 2 weeks apart) to request any relevant missing information. Data extraction will be conducted independently by two reviewers, and discrepancies checked for accurate extraction.

The data extraction grid with item definitions is available in Table 3 in Additional file 2.

### Quality assessment

The risk of bias and the quality of each study will be assessed using two checklists. For randomized trials, the risk of bias will be assessed with the Cochrane RoB 2.0 Tool [24]. For observational studies, we will use the ROBINS-I tool [25]. As the review does not intend to estimate a global measure of effect, no studies will be excluded based on the quality assessment. Quality assessment will only serve for analysis purposes and discussion of findings. This assessment will follow the same procedure as the data collection process. The quality assessment of each study will be done independently by two reviewers. Disagreements will be resolved by discussion between the reviewers or with a third reviewer as a referee.

### Assessment of reporting

Included studies will be classified according to the quality of their reporting. The Detailed Guidelines for Reporting on Latent Trajectory Studies (GRoLTS) [26] and the ESPACOMP Medication Adherence Reporting Guideline (EMERGE) [27] will be used to evaluate the studies. No study will be excluded during this step; instead, the reporting quality will be used for discussion purposes.

### Analysis

Data analysis will proceed in three phases. In the first phase, we will describe selected studies with simple descriptive statistics and classify them in a table under the health domain studied (e.g., cancer, cardiovascular disease), medications used, population, and the studies’ stated objective. In the second phase, we will describe the GBTM parameters and modeling options used to estimate medication adherence trajectories. Studies will also be classified according to the source of data for medication adherence, medication adherence measure, parameters used in GBTM, including the rationale behind parameter choice (e.g., statistics, the order of polynomials, number of groups) and criteria for selecting the best model (clinical characteristics, the minimal number of patients included, etc.).

In the third phase, we will perform classification and narrative synthesis. We will review the choice and modalities used to estimate the association between medication adherence trajectories and health-related outcomes, including source of data, nature, and definition of the health-related outcomes studied, statistical model used, effect measure used, and how medication adherence measure was used in the model. We will also summarize limitations and biases reported by the authors and attempts to mitigate them. Moreover, we will classify studies according to reporting quality and overall quality in the synthesis. As the review does not aim to estimate a measured effect, we will not conduct a meta-analysis and assess between-study heterogeneity.

## Discussion

GBTM method has grown in popularity in adherence research over the last 20 years [28]. The method and its applications, the macro-implementations in the software, are well established and developed in many disciplines, such as pharmacoepidemiology [29]. Most of the studies on GBTM in this field have primarily used trajectories to describe medication adherence over time and associated factors [30, 31]. Thus, their statistical models have mainly used trajectories as dependent variables. To our knowledge, two systematic reviews on latent class modeling approaches, including the GBTM method exist [32, 33]. However, they did not specifically examine medication adherence trajectories issued from GBTM as independent variables.

We will therefore provide a systematic synthesis of how associations between medication adherence trajectories estimated from the GBTM method, and health-related outcomes are studied and described associated challenges. While the GBTM method provides a more refined measure of medication adherence over time by identifying medication adherence trajectories, [34] it remains essential to study the association between these trajectories and health-related outcomes. Despite the growing use of GBTM in adherence research and the availability of statistical tools, there is still considerable heterogeneity in how researchers use this method to study health-related outcomes [35, 36]. This again leads to disparate and sometimes confusing ways of studying and reporting their results .

Moreover, assessing the association between medication adherence trajectories and health-related outcomes presents statistical challenges. Therefore, the groups identified with the GBTM method are probabilistic. The group assignment may be considered a 100% imputation, possibly resulting in a not-quantifiable uncertainty when inference about these groups is made through regression. Another concern is non-identifiability since GBTM imputes group membership on a not sufficiently general model, resulting in attenuated estimates of the relationship between trajectories and health-related outcomes. These problems are not specific to the GBTM method but to all latent class modeling [37].

However, modeling health outcomes according to medication adherence trajectories could help identify problematic groups to subsequently guide interventions and policies. It is, therefore, necessary to review how the method is used to model data and how results are reported.

The review will summarize the various strategies and methods used by authors to estimate the association between medication adherence trajectories and health-related outcomes. Special attention will be paid to study designs, model parameter specifications, and limitations. It will also document biases that could arise while using GBTM as an independent variable and how authors attempted to mitigate them. Moreover, the review will also describe the studies’ reporting quality with the two reporting guidelines specific to latent class analysis and adherence studies. Therefore, this review could represent the first crucial step towards developing a guide for using medication adherence trajectories estimated with GBTM to infer health-related outcomes.

## Supplementary Information

**Additional file 1.** PRISMA-P 2015 Checklist.

**Additional file 2. **Inclusion Criteria: **Table S1**: Inclusion criteria. Search strategy: **Table S2**: Search strategy. Data extraction grid: **Table S3**: Data extraction grid.

## Data Availability

Not applicable

## Abbreviations

EMERGE: ESPACOMP Medication Adherence Reporting Guideline
GBTM: Group-based trajectory modeling
GRoLTS: The Detailed Guidelines for Reporting on Latent Trajectory Studies
MPR: Medication possession ratio
PDC: Proportion of days covered
PRISMA: Preferred Reporting Items for Systematic Review and Meta-analysis
Prisma-p: Preferred Reporting Items for Systematic Review and Meta-Analysis Protocols
RoB 2.0: A revised tool to assess the risk of bias in randomized trials
ROBINS-I: Risk Of Bias in Non-randomized Studies - of Interventions
