# Supplementary material for: What methods are used to study the association between medication adherence trajectories, estimated with the group-based trajectory modeling (GBTM) method, and health-related outcomes?—a protocol for a systematic review
Source: Syst Rev. 2022 May 23;11:102. doi: 10.1186/s13643-022-01971-y (PMC9128283; doi:10.1186/s13643-022-01971-y)
Supplement: Supplementary file 2 — Additional file 2. Inclusion Criteria: Table S1: Inclusion criteria. Search strategy: Table S2: Search strategy. Data extraction grid: Table S3: Data extraction grid. [file 13643_2022_1971_MOESM2_ESM.docx]

Appendix B

# Inclusion Criteria

*Table 1: Inclusion criteria*

| **Inclusion** |  |
| --- | --- |
| **Medication adherence** | Any study about medication adherence, estimated with any kind of method whether direct (e.g., pill count or plasmatic measurement) or indirect (e.g., questionnaire,  adherence measure using medication database such as the proportion of days’ covered medication possessing ratio, medication gap). |
| **Group-based trajectory** | The group-based trajectory modeling a statistical methodology for modeling the evolution of medication adherence over time. This includes the based method described by Nagin et al. ^19^ and all its extensions. |
| **Adherence trajectory** | Adherence trajectory estimated with group-based trajectory as defined by Nagin et al. and considered as an **independent variable** in any statistic modeling. |
| **Health related outcomes** | Health-related outcomes any health results measured following an intervention or behavior (e.g., surgery, treatment etc.) that describe a consequence of disease, treatment, or event for an individual. These health-related outcomes can be symptoms, hospitalizations, death, patient’s quality of life, participation in activities, and social roles |
| **Domain** | Health |
| **Type of document** | Original article,  Excluded: Conference abstract, commentaries, letter to editors, review. They will be nevertheless checked for references |
| **Timeline** | No restriction |
| **Language** | No restriction, document in other languages than English will be translated and extracted |
| **Population** | No restriction |
|  |  |

# Search strategy

*Table 2: Search strategy*

| Strategy | Pubmed | Embase | Psyinfo |
| --- | --- | --- | --- |
| Treatment adherence | "treatment adherence and compliance"[MeSH Terms] OR "adheren*"[Title/Abstract] OR "adheren*"[Other Term] OR "complian*"[Title/Abstract] OR "complian*"[Other Term] OR "persisten*"[Title/Abstract] OR "persisten*"[Other Term] OR "nonadheren*"[Title/Abstract] OR "nonadheren*"[ Other Term] OR "noncomplian*"[Title/Abstract] OR "noncomplian*"[Other Term] OR "nonpersisten*"[Title/Abstract] OR "nonpersisten*"[Other Term] | **'patient compliance'**/exp OR **adheren***:ab,ti,kw OR non**adheren***:ab,ti,kw OR **complian***:ab,ti,kw OR non**complian***:ab,ti,kw OR **persisten***:ab,ti,kw OR non**persisten***:ab,ti,kw OR **'persistence':de** | Treatment Compliance/ OR complian*.ti,ab,id OR noncomplian*.ti,ab,idOR persisten*.ti,ab,idOR nonpersisten*.ti,ab,id OR adherenc*.ti,ab,id OR nonadherenc*.ti,ab,id |
| Compliance |  |  |  |
| Adherence |  |  |  |
| And |  |  |  |
| Group based trajectory modelling | Trajectories[TIAB]  OR Trajectory[TIAB]  OR GBTM[OT]  OR GBTM[TIAB] | 'trajectory analysis':de OR 'trajector* near/3 model*':ab,ti,kw OR 'gbtm':ab,ti,kw | Trajector*.ti,ab,id OR GBTM .ti,ab,id |
| Trajectory modelling |  |  |  |
|  |  |  |  |

| Strategy | Web of science | CINAHL | Cochrane database |
| --- | --- | --- | --- |
| Treatment adherence | ts=(adheren* or complian* or persisten* OR nonadheren* or noncomplian* or nonpersisten*) | MH medication compliance OR AB ( complian* or adheren* or persisten* ) OR AB ( noncomplian* or nonadheren* or nonpersisten* ) OR TI ( complian* or adheren* or persisten* ) OR TI ( noncomplian* or nonadheren* or nonpersisten* ) | [mh "Treatment Adherence and Compliance"] OR(adheren*):ti,ab,kw OR (nonadheren*):ti,ab,kw OR (complian*):ti,ab,kw OR (noncomplian*):ti,ab,kw OR (persisten*):ti,ab,kw OR (nonpersisten*):ti,ab,kw |
| Compliance |  |  |  |
| Adherence |  |  |  |
| And |  |  |  |
| Group based trajectory modelling | ts= (trajector* NEAR/3 model*) OR ts=(gbtm) | TI (trajector* N3 model*) or AB (trajector* N3 model*) OR TI gbtm OR AB GBTM | (trajector*):ti,ab,kw OR (GBTM):ti,ab,kw |
| Trajectory modelling |  |  |  |
|  |  |  |  |

# Data extraction grid

Table 3: Data extraction grid

| **Variables** | **Definition** | **Format (or example)** |
| --- | --- | --- |
| Identification | | |
| Id | Report the order number of the article assigned incrementally | 1 |
| Author | Report the name of the corresponding author as recorded in the article | name, surname |
| Mail | Report the corresponding author’s mail | xxxx@xxxx.xx |
| Year | Report the publication year of the article | yyyy |
| Country | Report the country where the study has been conducted |  |
| Title | Report the title of the article |  |
| Study design | Report the design of the study (Randomised trial, cohort study, case-control, other) |  |
| **Objectives** | | |
| Objectives | Report the objectives of the article as stated in the paper (copy and paste) |  |
| Medication, intervention, analysis object | | |
| Intervention | Report whether the study included an intervention to improve adherence | yes  no  unclear |
| Medication class | Report the medication or class of medication of interest | antidiabetic drugs  antihypertensive drugs  cardiovascular drugs  asthma drugs  oncology drugs  other, specify |
| Medication other | If other, specify the medication or class of medication of interest |  |
| Medication’s name | Report the name of all the medications mentioned |  |
| Prevalent or incident user | Report whether the participants are new users or prevalent users of the medication | prevalent user  incident user  both  unclear |
| Population disease | Report diseases of concern in the study |  |
| Population age group | Specify whether there is any description of age group in the study |  |
| Sample size | Report whether the sample size calculation was performed before the study | yes  no  unclear |
| Sample size calculation | Report whether the sample size calculation was performed considering the GBTM or the HRO | power for GBTM  power for the HRO  both  other |
| Sample size method | Report the method used to estimate the sample size |  |
| **Adherence** | | |
| Adherence measure: electronic monitoring caps (EMC) device | Report whether adherence is measured by an electronic device (EMC or similar device) | yes  no |
| Adherence measure: adherence questionnaire | Report whether an adherence questionnaire measures adherence | yes  no |
| Adherence measure: adherence questionnaire type | Report the adherence questionnaire used |  |
| Adherence measure: visual analog scale | Report whether adherence is measured using a visual analog scale | yes  no |
| Adherence measure: visual analog scale type | Report the visual analog scale used by the author |  |
| Adherence measured by drug concentration/metabolite levels (in the blood) | Report whether the study has measured adherence using drug concentration/metabolite levels | yes  no |
| Adherence measured by pill count | Report whether the study has measured adherence by pill count | yes  no |
| Adherence measure: medico-administrative database | Report whether the study computed adherence from a medico-administrative database | yes  no |
| Adherence measure:  claims database | Report whether the study used a medico-administrative database from prescription claims | yes  no  unclear |
| Adherence measure:  prescription database | Report whether the study used a medico-administrative database from physician prescriptions | yes  no  unclear |
| Adherence measure in database: type | Report adherence measure used in the medico-administrative database | PDC  MPR  medication gap  other, specify |
| Adherence measure in database: other | If other, report the name of the adherence measure used in the database |  |
| Adherence measure in database: definition | Report whether the study reports a clear description of the adherence measure used | yes  no  unclear |
| Adherence measure in database: definition | Report the definition of adherence measure as stated in the document (copy and paste) |  |
| Adherence measure: time frame | Report whether the authors have indicated the period of measurement of adherence | yes  no |
| Adherence measure: time frame definition | If yes, indicate the period of measurement of the adhesion (weekly, monthly, quarterly, etc.) |  |
| **GBTM** | | |
| GBTM: software | Report the software used for the GBTM |  |
| GBTM: software package | Report the package used |  |
| GBTM: time horizon | Report the time horizon used for adherence trajectories |  |
| GBTM: form of the likelihood function - report | Report whether the form of the likelihood function has been reported | yes  no |
| GBTM: form of the likelihood function | Specify the form of the likelihood function used | Poisson distribution  censored normal distribution  binary logit distribution  other  not applicable |
| GBTM: form of the likelihood function_other | If other, report the form of the likelihood function used |  |
| GBTM: Link function order | Report whether the link function order has been reported | yes  no |
| GBTM: Link function order – report | Report the link function order |  |
| GBTM: Link function order justification | Report whether the authors justified the choice of the link function order | yes  no |
| GBTM: number of groups tested | Report whether the author(s) has reported the number of groups tested | yes  no |
| GBTM: number of groups tested | If yes, report the number of groups tested |  |
| GBTM: number of groups selected | Report the number of groups selected |  |
| GBTM: number of groups selected justification report | Report whether the authors have provided a rationale for the choice of the number of groups selected | yes  no |
| GBTM: number of groups selected justification report | If yes, report the statement for the rationale behind the choice of the number of groups |  |
| GBTM- Best fit parameters choice model in consideration | | |
| GBTM: Best fit parameters AIC | Report whether the authors have considered the AIC in model choice | yes  no |
| GBTM: Best fit parameters AIC_all_models | Report whether the authors have reported the AIC for all different models considered | yes  no |
| GBTM: Best fit parameters AIC_best_model | Report whether the authors have reported the AIC for the model selected | yes  no |
| GBTM: Best fit parameters BIC | Report whether or not the authors have considered the BIC in choice of the model | yes  no |
| GBTM: Best fit parameters BIC_all models | Report whether the authors have reported the BIC for all different models considered | yes  no |
| GBTM: Best fit parameters BIC_best model | Report whether the authors have reported the BIC for the model selected | yes  no |
| GBTM: Minimum size per group | Report whether the authors have reported the minimum size per group in the choice of the model | yes  no |
| GBTM: Best-fit -Average Posterior Probability of Assignment (APPA) | Report whether the authors have considered the Average Posterior Probability of Assignment (APPA) in choice of the model | yes  no |
| GBTM: Best-fit -APPA all models | Report whether the authors have reported the APPA for the different models in consideration | yes  no |
| GBTM: Best-fit -APPA best model | Report whether the authors have reported the APPA for the model selected | yes  no |
| GBTM: Best-fit -Odds of Correct Classification (OCC) | Report whether the authors have considered the Odds of Correct Classification (OCC) in choice of the model | yes  no |
| GBTM: Best-fit -OCC- all models | Report whether the authors have reported the OCC for the different models in consideration | yes  no |
| GBTM: Best-fit -Odds of Correct Classification-best model | Report whether the authors have reported the OCC for the model selected | yes  no |
| GBTM: Estimated Group Probabilities versus the Proportion of the Sample Assigned to the Group | Report whether the authors have considered the Group Probabilities versus the Proportion of the Sample Assigned to the Group in the choice of the model | yes  no |
| GBTM- Best-fit- Estimated Group Probabilities versus the Proportion of the Sample Assigned to the Group-all models | Report whether the authors have considered the Group Probabilities versus the Proportion of the Sample Assigned to the Group for the different models in consideration | yes  no |
| GBTM- Best-fit- Estimated Group Probabilities versus the Proportion of the Sample Assigned to the Group-best model | Report whether the authors have considered the Group Probabilities versus the Proportion of the Sample Assigned to the Group for the model selected | yes  no |
| GBTM Best fit: Confidence Intervals for Group Membership Probabilities | Report whether the authors report the confidence intervals for group membership probabilities | yes  no |
| GBTM Best fit: Tool for fit criteria assessment | Report whether the authors have used a fit criteria assessment tool for model selection | yes  no  unclear |
| GBTM Best fit: tool for fit criteria assessment | If yes, report the name of the tool |  |
| Analysis_Sensibility_GBTM | Report whether the authors have conducted any sensibility analysis for GBTM | yes  no |
| **Missing Data** | | |
| Missing Data | Report whether the authors considered missing data | yes  no  unclear |
| Missing data adherence | If yes, report how authors managed missing data in GBTM | exclusion  imputation  other |
| Missing data adherence_other | If other, specify |  |
| Missing data HRO | If yes, report how authors managed missing data in HRO modeling | exclusion  imputation  other |
| Missing data HRO_other | If other, report how authors managed missing data in HRO modeling |  |
| Censoring | Report whether the authors took into account censored data | yes  no  unclear |
| Censoring GBTM | If yes, report how authors managed censoring data in GBTM |  |
| Censoring HRO | If yes, report how authors managed censoring data in HRO |  |
| Health-related outcomes (HRO) | | |
| HRO: definition | Report whether the study report a clear definition of the HRO | yes  no |
| HRO: definition_report | Report the definition of the outcomes, including how they were measured |  |
| HRO: outcomes data sources | Report the data source of the outcomes |  |
| HRO: nature | Report whether the study report a clear definition of the HRO variable | continuous  dichotomic  categorial  ordinal |
| HRO: time_related | Report whether the HRO defined in the article is time-related (e.g., number of hospitalizations per month), an instant screen shoot (e.g., cross-sectional), health measure (e.g., quality of life), or definitive (e.g., death) | time-related  instant screenshot  definitive |
| HRO: modelling | Report whether the authors have included the HRO variable in the modelling of GBTM | yes  no |
| HRO: model | Report the statistical method used to estimate the association between adherence trajectories and HRO | modeled in GBTM  cox modeling  logistic regression  linear regression  dual trajectory modeling  other |
| HRO: model_other | If other, report the model used to estimate the association between adherence trajectories and HRO |  |
| HRO: model_rationale | Report whether the authors provide a rationale for the choice of the model | yes  no |
| HRO: model_rationale_report | If yes, report the rationale of the model choice |  |
| HRO:  time_lag | Report whether the outcomes were measured immediately after the end of the trajectories or not (yes =distal time, dual = same time of trajectory, no = no time lag) | yes  dual  no  unclear/not specified |
| HRO: time_lag_definition | Report the time lag (in months) |  |
| HRO: time_lag_rationale | Report whether the authors have provided a rationale for the definition of the time lag used | yes  no |
| HRO:  time_lag_rationale | If yes, report the rationale used |  |
| HRO: Adherence trajectories used | Report how trajectories have been used in the model (e.g., trajectory groups as a variable, inverse probability weighting, e.tc) |  |
| Analysis_Sensibility_HRO | Report whether the authors have conducted any sensibility analysis for the HRO modelling | yes  no |
| **Limitations** | | |
| Confounding by indication | Report whether the authors have reported confounding by indication as a possible limitation of the study | yes  no |
| Time-dependent confounding | Report whether the authors have reported time-dependent confounding as a possible limitation of the study | yes  no |
| Healthy user/adherer effect | Report whether the authors have reported healthy user/adherer effect as a possible limitation of the study | yes  no |
| Protopathic bias | Report whether the authors have reported protopathic bias as a possible limitation of the study | yes  no |
| Depletion of susceptible | Report whether the authors have reported depletion of susceptible as a possible limitation of the study | yes  no |
| Time-related bias | Report whether the authors have reported time-related bias as a possible limitation of the study | yes  no |
| Immortal time bias | Report whether the authors have reported immortal time bias as a possible limitation of the study | yes  no |
| Immeasurable time bias | Report whether the authors have reported immeasurable time bias as a possible limitation of the study | yes  no |
| Time-window bias | Report whether the authors have reported time-window bias as a possible limitation of the study | yes  no |
| Time-lag bias | Report whether the authors have reported time-lag bias as a possible limitation of the study | yes  no |
| **Others** | | |
| Conflict of interest | Report whether the authors have reported conflict of interest | yes  no |
